# Quantitative evaluation of anisometropic amblyopia treatment efficacy by coupling multiple visual functions via CRITIC algorithm

**DOI:** 10.1186/s12886-023-02898-1

**Published:** 2023-04-18

**Authors:** Ying Zhi, Min Cai, Rui Du, Ying Qiao, Xiaowei Zheng, Guanghua Xu, Li Yan, Dianpeng Wu

**Affiliations:** 1 Department of Ophthalmology, First Affiliated Hospital of Northwest University, Xi’an, China; 2 Department of Ophthalmology, Xi’an No. 1 Hospital, Xi’an, China; 3 School of Mathematics, Northwest University, Xi’an, China; 4 School of Mechanical Engineering, Xi’an Jiaotong University, Xi’an, China; 5 National Engineering Research Center for Healthcare Devices, Guangzhou, China

**Keywords:** Amblyopia, Treatment efficacy, Coupling method, CRITIC algorithm

## Abstract

**Background:**

The evaluation of amblyopia treatment efficacy is essential for amblyopia prevention, control, and rehabilitation.

**Methods:**

To evaluate the amblyopia treatment efficacy more precisely and quantitatively, this study recorded four visual function examination results, i.e., visual acuity, binocular rivalry balance point, perceptual eye position, and stereopsis before and after amblyopia treatment.

**Results:**

We found that all these four results had a significant difference between before and after treatment, and the relationship between visual acuity improvement and the difference of BRBP, PEP, and stereoacuity cannot show a fitting correlation regarding the widely used index of visual acuity as the standard of treatment efficacy. By using the Criteria Importance Through Inter-criteria Correlation (CRITIC) method, a more comprehensive and quantitative index by coupling the selected four indexes with objective weights was obtained for further training efficacy representation, and the validation dataset also showed a good performance.

**Conclusions:**

This study proved that our proposed coupling method based on different visual function examination results via the CRITIC algorithm is a potential means to quantify the amblyopia treatment efficacy.

## Introduction

Amblyopia is one of the common visual disorders caused by abnormalities in the visual system during early childhood with a prevalence of 3%-5% [1–3]. It is mainly shown that the best corrected visual acuity is lower than that of the normal age-matched eye without obvious ocular pathology [4]. Previous studies have shown that the common predisposing factors for amblyopia contain strabismus, refractive error, and form deprivation [5, 6].

The most disrupting consequence of amblyopia is interocular suppression and functional loss in the amblyopic eye [7], which mainly manifests the impairments in visual acuity, contrast sensitivity, stereoacuity, and other losses, e.g., disrupted eye-hand coordination, disturbed oculomotor functions, and mislocalization of visual stimuli [8]. Therefore, to realize the accurate detection of amblyopia from all aspects, a series of visual function diagnoses is necessary, e.g., visual acuity [9], perceptual eye position (PEP) [10], and stereoacuity [11], etc.

Perceptual learning technology, especially push–pull perception, provides an effective means for amblyopia treatment [12]. Push–pull perception can restore the function of amblyopia, e.g., improvement of visual acuity and stereoacuity, by adjusting the intensity of visual stimuli in two eyes [13]. Hence, the fine quantification of amblyopia treatment efficacy is essential in amblyopia accurate treatment and training program adaptation [14, 15]. Meanwhile, since amblyopia can cause many visual disorders, the traditional single visual function examination, like the visual acuity test, does not meet the need for quantification of amblyopia treatment efficacy [16]. Therefore, coupling multiple classical visual function test results provides an alternative means for quantitative assessment of amblyopia treatment. However, till now, little is known about whether the coupling information of several related visual function test results can improve the precision of the assessment of amblyopia treatment efficacy.

In this study, we aim to combine the results of several visual function examinations related to amblyopia to obtain a more precise expression of amblyopia treatment efficacy. Here, first, four visual functions, i.e., visual acuity, binocular rivalry balance point (BRBP), PEP, and stereoacuity, were chosen and their results were compared before and after anisometropic amblyopia treatment. Then, the relationship between visual acuity improvement and the difference in BRBP, PEP, and stereoacuity was analyzed to find whether there is a fitting correlation. Next, the difference of these four visual functions was coupled by weighting to acquire a more precise coupling index by using the Criteria Importance Through Inter-criteria Correlation (CRITIC) algorithm. Finally, this new index was used to evaluate the amblyopia treatment efficacy more precisely.

## Methods

### Subject

Forty-six children (ages 4–12 years, eighteen females) with anisometropic amblyopia, recruited from the Xi’an No.1 Hospital (Xi’an, China), participated in this experiment. For each subject, there is only one amblyopic eye, and the other fellow eye is normal, that is, they are all unilateral amblyopia. All the participants wore their best-corrected optical glasses during the experiment.

### Experimental procedure

For each subject, before the amblyopia training, four visual function examination tasks, i.e., visual acuity, BRBP, PEP, and stereopsis, were required to be accomplished. Next, an amblyopia treatment was carried out for half a year to one year to reach a relatively good effect. Then, these four examination tasks were done again.

### Visual acuity examination

Visual acuity, one of the most basic visual functions, is a measure of the ability to recognize small details in the center of the visual field with precision. In this study, visual acuity was assessed by a standard logarithmic visual acuity chart [17], which has been a widely used visual acuity test standard for over 20 years, especially in China. The equal length of the three lines of E was used as optotype, and the measuring range is from 0.1 to 2.0 at 14 steps in the decimal recording method, corresponding to 1.0 to -0.3 logMAR with 0.1 logMAR between the two steps.

### Cerebral visual function examination

For the cerebral visual function examination of BRBP, PEP, and stereopsis, in this study, a cerebral visual functions evaluation system invented by the National Engineering Research Center for Healthcare Devices (Guangzhou, China) was used with the hardware including a computer host, a 23-inch 3D display (LG 2343p, Seoul, South Korea) with a resolution of 1920 × 1080 and a refresh rate of 120 Hz, and 3D polarized glasses [18, 19]. The test demo was programmed by MATLAB (MathWorks, Natick, United States).

### Binocular rivalry balance point

**Fig. 1:**
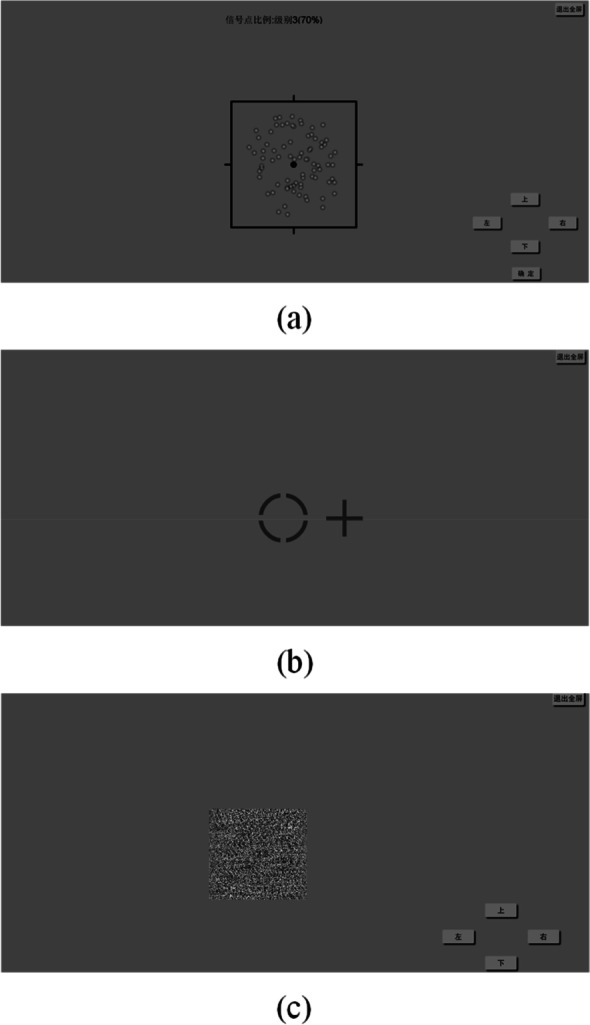
Examples of cerebral visual function examinations. **a** BRBP examination. **b** PEP examination. **c** Stereoacuity examination

Hess et al. [20, 21] proposed and designed the concept and detection method of the binocular rivalry balance point, and then evaluated binocular vision, which reflects the competitive relationship and reciprocal inhibition between two eyes. In this study, as shown in Fig. 1(a), the signal points and noise points can be presented separately in two eyes under the condition of dichoptic viewing. The signal points moved toward a direction at a uniform speed, while the noise points moved in a disorderly form. The subjects were required to distinguish the movement direction of signal points. Then, the proportion of signal and noise points was changed until the observing eye of signal points cannot distinguish the movement direction under the interference of noise points from another eye, that is, the balance point is obtained. The proportion of signal and noise points is divided into eight levels: level 1 is 100% signal points with no noise points; level 2 is 85% signal points with 15% noise points; level 3 is 70% signal points with 30% noise points. After that, the ratio of noise points is increased by 10% and the ratio of signal points is decreased by 10% at each level until the ratio of signal points is 20% and the ratio of noise points is 80% at level 8. Each level was tested 3 times, and the correct level was passed until reaching the balance point. Finally, the levels of the proportion of signal and noise points at the balance point for two eyes were recorded respectively.

#### Perceptual eye position

PEP is a measure of the deviation of the perceptual position from the actual position, which can reflect the visual cortex’s separation control of the eye position [22, 23]. The larger deviation of PEP corresponds to the worse control of eye position from the visual cortex. In this study, for the PEP test, first, subjects were asked to sit 80 cm away from the polarized display. Next, as shown in Fig. 1(b), under the situation of dichoptic viewing, the right eye can see a “circle” fixed at the center of the screen, and in the meantime, the left eye can see a “cross” whose position can be controlled by the computer mouse. Then, subjects were asked to move the cross to the center of the circle from the horizontal or vertical direction. Finally, the deviation of horizontal and vertical PEP in degree units can be calculated. In addition, the size of the circle was 0.4 × 0.4º, and the size of the cross was 0.33 × 0.33º.

#### Stereoacuity

Stereoacuity is used to describe the ability to discriminate depth based on binocular retinal disparity, and this binocular visual function is associated with anisometropic type and magnitude [24, 25]. In this study, a 3D zero-order random-dot test was used to obtain stereoacuity. As shown in Fig. 1(c), for each trial, there was an “E” with a random direction at the center of the gray background composed of random and stationary dots when observed by polarized glasses. In total, the stereoacuity examination contained four trials corresponding to four stereoacuity levels of 400, 300, 200, and 100 s of arc. Subjects were required to discriminate the direction of “E” of each trial, and the final stereoacuity result was recorded as the lowest second of arc level. Besides, if the directions of all four trials cannot be discriminated correctly by one subject, the subject’s stereoacuity was completely impaired. For convenience, this study used five levels of 4, 3, 2, 1, and 0 to represent the four stereoacuity levels of 100, 200, 300, 400, and non-stereoacuity.

### Amblyopia treatment

The personalized training system of push–pull and disinhibition model based on a virtual reality system also provided by the National Engineering Research Center for Healthcare Devices (Guangzhou, China) was adopted for amblyopia treatment. According to the polarized 3D technology, a stimulation for binocular balance and a disinhibition model can be established [13, 26]. Subjects were asked to perform personalized push–pull model training twice a day each time lasting 20 min, and the training program was adjusted every three months according to the patient's visual state. The amblyopia treatment for each subject lasted half a year to one year.

### Coupling method

In this study, we aimed to combine the results of several visual function tests to propose a novel index to reflect the efficacy of amblyopia treatment more precisely and comprehensively. Here, as mentioned above, four single indexes, i.e., visual acuity, BRBP, PEP, and stereoacuity, were obtained. Then, a coupling algorithm, named the CRITIC algorithm [27], was used to solve the problem of determining weights for these four indexes. The CRITIC algorithm is an objective weighting method based on two indicators of the data itself: contrast intensity representing each factor and the conflict between two factors, considering both the trend of a single index and the correlation among different indexes [28, 29]. For the CRITIC algorithm, the main steps are as follows.

i Establish a data matrix based on the initial data:
  1 $${\varvec{X}}={\left({x}_{ij}\right)}_{m\times n}$$
  where ${x}_{ij} (i=1, 2,\dots ,m;j=1, 2,\dots , n)$ is the original data corresponding to the *j*-th indicator of the *i*-th sample.
ii Using the Z-score method, standardize the original data matrix above:
  2 $${x}_{ij}^{*}=\frac{{x}_{ij}-{\overline{x} }_{j}}{{s}_{j}}$$
  3 $${\overline{x} }_{j}=\frac{1}{m}\sum_{i=1}^{m} {x}_{ij}$$
  4 $${s}_{j}=\sqrt{\frac{1}{m-1}\sum_{i=1}^{m} {\left({x}_{ij}-{\overline{x} }_{j}\right)}^{2}}$$
  where ${\overline{x} }_{j}$ is the average of the *j*-th indicator, ${s}_{j}$ is the standard deviation of the *j*-th indicator, and ${{\varvec{X}}}^{*}={\left({x}_{ij}^{*}\right)}_{m\times n}$ is the standardized matrix.
iii Calculate the variation coefficient of each indicator:
  5 $${v}_{j}=\frac{{s}_{j}}{{\overline{x} }_{j}}$$
  where ${v}_{j}$ is the variation coefficient of the *j*-th indicator.
iv Calculate the correlation coefficient matrix ${\varvec{R}}={\left({r}_{kl}\right)}_{n\times n}$ of matrix ${{\varvec{X}}}^{*}$:
  6 $${r}_{kl}=\frac{\sum_{i=1}^{m} \left({x}_{ik}^{*}-{\overline{x} }_{k}^{*}\right)\left({x}_{il}^{*}-{\overline{x} }_{l}^{*}\right)}{\sqrt{\sum_{i=1}^{m} {\left({x}_{ik}^{*}-{\overline{x} }_{k}^{*}\right)}^{2}}\sqrt{\sum_{i=1}^{m} {\left({x}_{il}^{*}-{\overline{x} }_{l}^{*}\right)}^{2}}}$$
  where ${r}_{kl}$ denotes the correlation coefficient between the *k*-th indicator and *l*-th indicator.
v Identify the independence coefficient ${\eta }_{j}$ of each indicator to assess the degree of correlation among different indicators:
  7 $${\eta }_{j}=\sum_{k=1}^{n} \left(1-\left|{r}_{kj}\right|\right), j=\mathrm{1,2},\dots ,n$$
vi Calculate the total volume of information for each indicator:
  8 $${D}_{j}={v}_{j}{\eta }_{j}, j=1, 2, 3,\dots ,n$$
vii Determine the weight of each indicator:
  9 $${\omega }_{j}=\frac{{D}_{j}}{\sum_{j=1}^{n} {D}_{j}}, j=1, 2, 3,\dots ,n$$

### Statistical analysis

The paired *t*-test was used to compare the four single indexes, i.e., visual acuity, BRBP, PEP, and stereoacuity, between before and after amblyopia treatment.

## Results

### Qualitative analysis of each examination results

#### Visual acuity

**Fig. 2:**
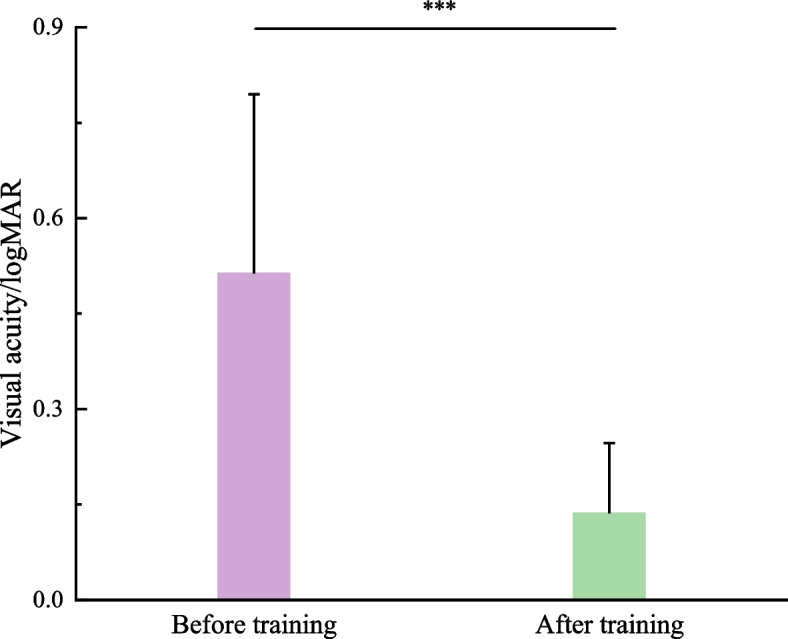
Comparison of visual acuity of amblyopic eyes before and after treatment

The subjects who participated in this study were all unilateral anisometropic amblyopia. As shown in Fig. 2, paired *t*-test found that the visual acuity of amblyopic eyes improved significantly after push–pull perception training (*t*_45_ = 9.766, *P* < 0.001), indicating that visual acuity can be an index to evaluate the training efficacy.

#### Binocular rivalry balance point

**Fig. 3:**
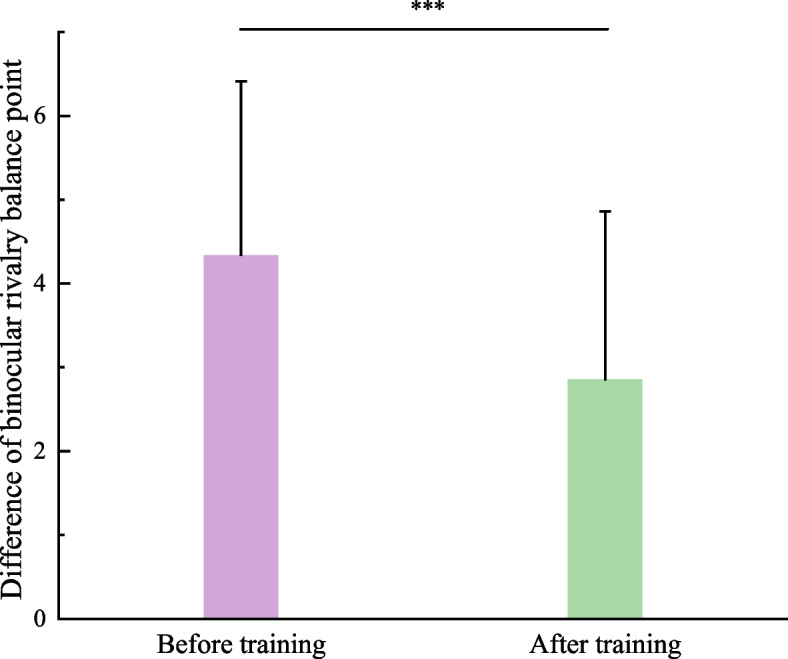
Comparison of difference of BRBP between two eyes before and after treatment

Subsequently, the difference in BRBP between two eyes for each subject was obtained. As shown in Fig. 3, paired *t*-test found that the BRBP difference of two eyes decreased significantly after push–pull perception training (*t*_45_ = 4.965, *P* < 0.001), also indicating that the difference in BRBP can be an index to evaluate the training efficacy.

#### Perceptual eye position

**Fig. 4:**
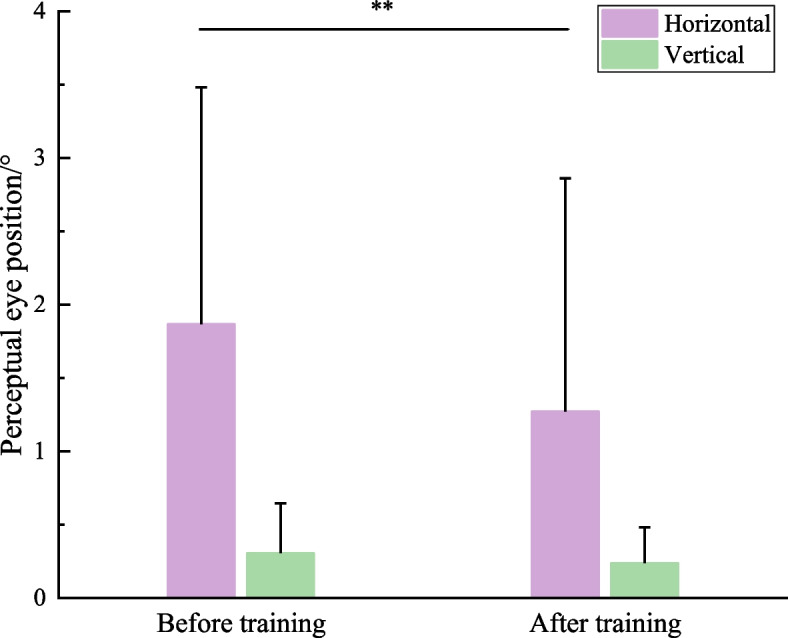
Comparison of horizontal and vertical PEP before and after treatment

As shown in Fig. 4, the horizontal and vertical PEP before and after training was obtained, respectively. Then, paired *t*-test found that the horizontal PEP decreased significantly after training (*t*_45_ = 3.094, *P* = 0.003), and the vertical had a slightly but non-significantly decreasing trend (*t*_45_ = 1.520, *P* = 0.136), demonstrating that the horizontal PEP can be regarded as an index to evaluate the training efficacy.

#### Stereoacuity

**Fig. 5:**
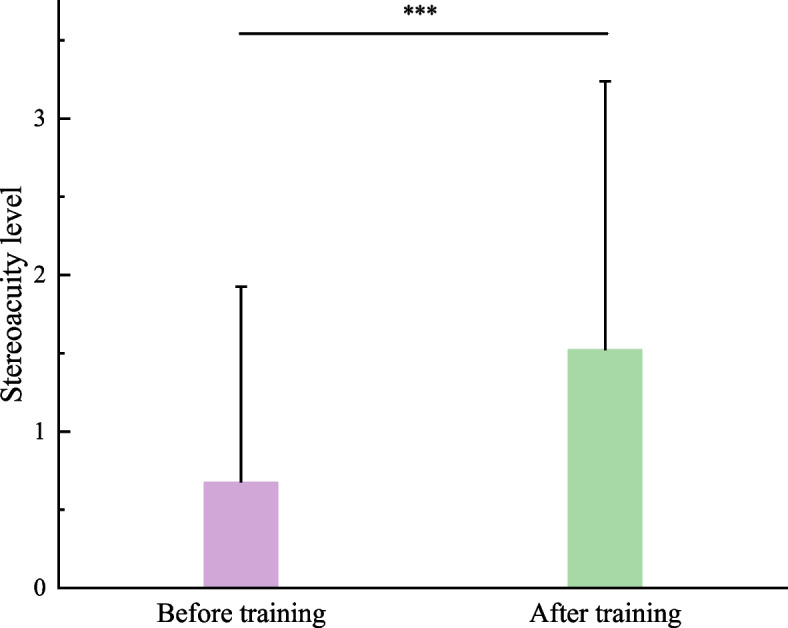
Comparison of stereoacuity before and after treatment

Likewise, the stereoacuity before and after training was also obtained, as shown in Fig. 5. Paired *t*-test found that the stereoacuity increased significantly after training (*t*_45_ = -4.548, *P* < 0.001), demonstrating that the stereoacuity can be regarded as an index to evaluate the training efficacy.

#### Correlation analysis among four examination results

Here, the degree of visual acuity improvement before and after training was regarded as the standard of training efficacy. Therefore, the correlation analysis was carried out to analyze the relationship of the difference in three cerebral visual function examinations between before and after training and visual acuity improvement, respectively.

**Fig. 6:**
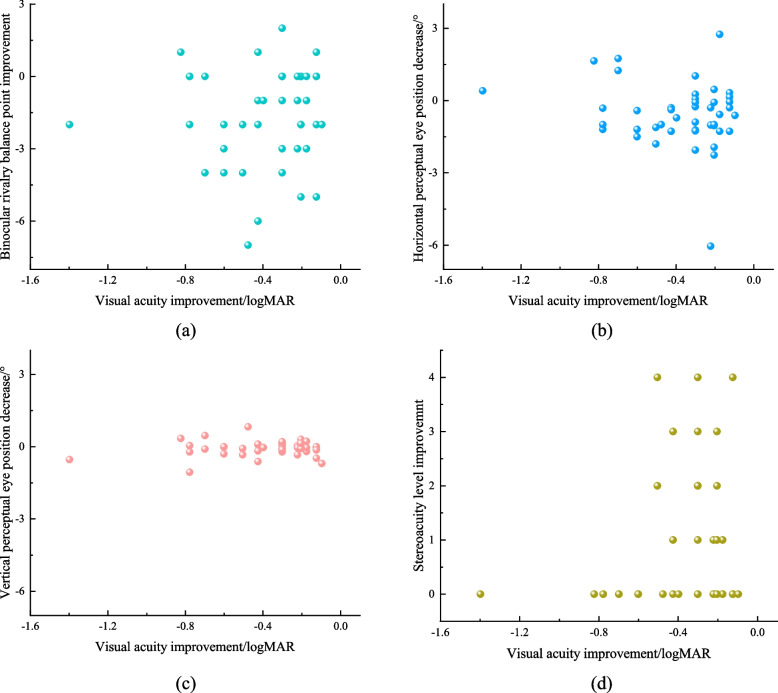
Relationship between visual acuity improvement and difference of BRBP, PEP, and stereoacuity before and after treatment, respectively. **a** BRBP. **b** Horizontal PEP. **c** Vertical PEP. **d** Stereoacuity

As shown in Fig. 6, all the relationships between visual acuity improvement and the difference in BRBP, PEP, and stereoacuity before and after training cannot show a fitting correlation, although the qualitative analysis above showed a consistent or contrary trend. In fact, the relationship between any two types of results cannot show a fitting correlation, either. The reason for this phenomenon may be that these four visual function examination results reflect the different aspects of the vision system, which may all be related to the degree of amblyopia and treatment efficacy. Therefore, this result also validated our hypothesis that a single visual function result cannot reflect the visual function state of amblyopia, and coupling results are needed.

#### Coupling method

According to the qualitative analysis above, the difference of four visual function indexes between before and after treatment, i.e., visual acuity, BRBP, horizontal PEP, and stereoacuity, were chosen to obtain a coupling index to assess the efficacy of amblyopia treatment more precisely. The correlation coefficient and weight of each index were determined by the CRITIC algorithm. As shown in Tables 1 and 2, the main parameter of the CRITIC algorithm was obtained, and the weights for indexes of visual acuity, BRBP, horizontal PEP, and stereoacuity were 0.2154, 0.2575, 0.1724, and 0.3547, respectively, with the stereoacuity having a maximal weight, showing that the change of stereoacuity can reflect the efficacy of amblyopia treatment to the great extent.

**Table 1:** Correlation coefficient between indexes of visual acuity, BRBP, horizontal PEP, and stereoacuity

Indexes	Visual acuity	BRBP	Horizontal PEP	Stereoacuity
Visual acuity	1.0000	0.1004	-0.1891	-0.1906
BRBP	0.1004	1.0000	0.0722	-0.1537
Horizontal PEP	-0.1891	0.0722	1.0000	0.0408
Stereoacuity	-0.1906	-0.1537	0.0408	1.0000

**Table 2:** Basic parameters of the CRITIC algorithm

Indexes	Mean ${\overline{x} }_{j}$	Standard deviation ${s}_{j}$	Variation coefficient ${v}_{j}$	Weights ${\omega }_{j}$
Visual acuity	-0.38	0.26	-1.46	0.2154
BRBP	-1.48	2.02	-0.73	0.2575
Horizontal PEP	-0.60	1.31	-0.46	0.1724
Stereoacuity	0.85	1.26	0.67	0.3547

To validate the reliability of the coupling method, new validation data from eight additional subjects were introduced, as shown in Table 3. For subject S1, the visual acuity improved a lot from 1.00 to 0.10 logMAR, the horizontal PEP improved from 1.2° to 0.64°, the BRBP of the left eye also improved from level 4 to level 7, and stereoacuity improved from 400″to 200″, demonstrating that the treatment efficiency of subject S1 was pretty good. Hence, the coupling index of subject S1 was slightly high corresponding to 0.5210. For subject S4, both the visual acuity improvement and stereoacuity improvement were lower than that of subject S1, and the coupling index was also lower. For subject S5, although the four visual function single indexes after training were not much different from that of subject S4, the coupling index of subject S5 was lower than S4, since the initial visual function status of S4 was worse than S5.

**Table 3:** Information of validation data and their coupling index

Subject	Before training					After training					CI
	VA	H-PEP	BRBP-R	BRBP-L	SA	VA	H-PEP	BRBP-R	BRBP-L	SA	
S1	1.00	1.2	8	4	1	0.10	0.64	8	7	3	0.5210
S2	0.60	1.5	7	3	0	0.10	0.18	8	6	1	0.3502
S3	0.60	0.5	2	8	0	0.10	0.32	6	8	0	0.3002
S4	0.80	0.58	8	1	0	0.20	0.36	8	6	0	0.3468
S5	0.80	0.48	6	5	0	0.10	0.38	8	6	0	0.1897
S6	0.20	0.42	5	8	0	0.10	0.14	7	8	0	0.1784
S7	0.50	1.46	3	4	4	0.15	0.8	8	6	4	0.1389
S8	0.40	0.28	4	8	0	0.10	0.1	6	8	0	0.2066

*VA* Visual acuity, *H-PEP* Horizontal PEP, *BRBP-R* BRBP of right eye, *BRBP-L* BRBP of left eye, *SA* Stereoacuity, *CI* Coupling index

## Discussion

This study proved that the coupling of the multi-index of visual function difference related to amblyopia before and after treatment can provide a quantitative means to evaluate amblyopia treatment efficacy. Data from forty-six subjects were included in the statistics of this study with pretty good results. The coupling weights for the four index difference before and after treatment of visual acuity, BRBP, horizontal PEP, and stereoacuity were 0.2154, 0.2575, 0.1724, and 0.3547, respectively. In fact, when adding to the eight validation data, the weights have changed slightly to 0.2221, 0.2643, 0.1633, and 0.3504 for visual acuity, BRBP, horizontal PEP, and stereoacuity, demonstrating that more data is needed for the standard establishment of coupling weights.

The reason why the difference of four visual function indexes between before and after treatment rather than the post-treatment indexes was chosen to calculate the coupling index was that the training efficacy was not only related to post-treatment visual status but also the visual status before treatment. For example, if a normal subject participated in this amblyopia treatment, his visual function after treatment is also normal, and we cannot prove his treatment is the best when only using the post-treatment visual function results. In addition, the coupling index of a patient with mild amblyopia will not be very high when completely cured, such as S5 in Table 3. Hence, the post-treatment visual status and the difference in visual status between before and after treatment may be considered together to obtain a more comprehensive index in future work.

Monocular deficits and interocular suppression are two main mechanisms of the formation of amblyopia [30, 31]. The push–pull perception can improve both the monocular and binocular visual functions [32]. As one of the vision perceptual learning, push–pull perception can activate visual signal pathways and improve the signal processing ability of the nervous system by taking advantage of the plasticity and transfer characteristics of the cerebral nervous system [12]. By enhancing the noise signal of the fellow eye and presenting stimulus signals to the amblyopic eye, the suppression from the fellow eye to the amblyopic eye can be reduced, so as to achieve the purpose of amblyopia treatment [33].

Amblyopia is a neurodevelopmental disorder caused by complicated factors [34], which also affects a series of visual functions. Yet, a single visual function only reflects a type of visual representation, causing that a single visual function cannot fully reflect amblyopia. Hence, effective synthesis of results from multiple visual function tests is theoretically potential to obtain a more precise result of amblyopia degree and state. In fact, the corresponding relation is also shown in other vision disorders. For example, glaucoma, a heterogeneous group of optic neuropathies, is often accompanied by visual acuity loss, elevated intraocular pressure, visual field defect [35], etc. Hence, effective synthesis of related visual function results provides a potential precise method for different visual defects.

Compared to the subjective psychophysical examinations, the electrophysiology methods, e.g., electroretinogram (ERG), electrooculogram (EOG), and electroencephalogram (EEG), offer a more objective and direct way to evaluate visual function [36], especially for the preverbal children, the mentally disabled, and malingerers [37]. In addition, some previous studies have proved that visual evoked potentials (VEPs), one of the widely used EEG patterns, provide an objective and quantitative means for the assessment of interocular suppression in amblyopia and even training efficacy [16, 38, 39]. Hence, it is very essential to promote the method of electrophysiological examination of visual function or visual system status for the determination of the degree of amblyopia and treatment efficacy.

Some limitations also needed to be weighed in this study. First, although the cerebral visual functions evaluation system used in this study was proven to be reliable in previous studies and was convenient and efficient to assess various visual functions [40–42], the gold standard for visual function evaluation may promote this study to be more widely used. Second, the amblyopia treatment is a very complex visual neuroplastic process, which is related to many factors, such as intervention age [43], training duration [44], training cooperativeness [45], amblyopia severity, etc., and future studies can compare the treatment efficacy at various factor levels to build a more complete model and refined index to quantify amblyopia treatment efficacy.

## Conclusion

In this study, firstly, we analyzed the multi-index difference of visual acuity, BRBP, PEP, and stereoacuity before and after amblyopia treatment qualitatively and obtained the indexes with significant change. Secondly, regarding the widely used index of visual acuity as the standard training efficacy, the relationship between visual acuity improvement and the difference of BRBP, PEP, and stereoacuity cannot show a fitting correlation, which validated our hypothesis that the coupling index is needed. Finally, using the CRITIC algorithm, the coupling weights of the selected four indexes were obtained for further training effect representation.

## Data Availability

The data generated and/or analyzed during this study are not publicly available due to legal/ethical reasons but are available from the corresponding author on reasonable request.
